# The current status of invasive alien insect species in South Korea

**DOI:** 10.3897/BDJ.10.e81941

**Published:** 2022-07-13

**Authors:** Dayeong Kim, Min-Ji Lee, Heejo Lee, Young-Gyu Ban, Dong Eon Kim

**Affiliations:** 1 Invasive Alien Species Team, Division of Ecological Threat Management, Conservation Research Bureau, National Institute of Ecology, Seocheon 33657, Republic of Korea Invasive Alien Species Team, Division of Ecological Threat Management, Conservation Research Bureau, National Institute of Ecology Seocheon 33657 Republic of Korea

**Keywords:** ecosystem-disturbing species, nationwide survey, natural ecosystem

## Abstract

We investigated the identity and distribution of the invasive alien insect species inhabiting Korean ecosystems, targeting 3,249 locations in nine regions between 2015 and 2018. In natural ecosystems, we identified 63 species in 43 families and nine orders of invasive alien insect species, respectively. We observed that the order Hemiptera exhibited the highest species diversity with 20 species. Gyeonggi-do was where the highest number of invasive alien insect species were identified (45 species). Species richness analysis revealed that Jeju-do showed the highest Dominance Index (0.8), whereas Gyeongsangnam-do had the highest Diversity Index (2.8). *Corythuchamarmorata* (Hemiptera: Tingidae), *Lycormadelicatula* (Hemiptera: Fulgoridae), *Ophraellacommuna* (Coleoptera: Chrysomeridae), *Metcalfapruinosa* (Say) (Hemiptera: Flatidae) and *Pochaziashantungensis* (Hemiptera: Ricaniidae) were distributed in more than 300 locations of the country. Invasive alien insect species inhabited the roadsides (31.3%), farmlands (18.3%) and parks (16.6%). In this study, we list the invasive alien insect species in Korean ecosystems and provide a basis for selecting primary management target species.

## Introduction

Increasing cross-border exchanges and international trade are contributing to the rapid introduction of invasive alien species (IAS) globally (Hulme 2021), which, together with the destruction of natural habitats, have been suggested to be one of the most important factors that threaten biodiversity. At the 10^th^ meeting for the Convention on Biological Diversity, 20 global goals (i.e. the 20 Aichi targets) to be achieved by 2020 were suggested for preserving biodiversity. Amongst these, the 9th goal states that the introduction and settlement of invasive alien species should be prevented by identifying their introduction routes and destroying them.

An invaded ecosystem may not have predators or competitors that would limit the growth of the invasive alien insect species (Čuda et al. 2015). Alternatively, ecosystems disturbed by human activities may be susceptible to IAS (Venette and Hutchison 2021). Furthermore, invasive alien insect species typically reproduce and grow rapidly, migrate easily, adapt their physiology to the new environment and survive on diverse food and various environments (Čuda et al. 2015). Collectively, these characteristics favour their wide spread in a certain region, damaging the ecosystem (Secretariat of the Convention on Biological Diversity 2010).

The unintentional introduction of IAS can occur via ships or on freights (Kang et al. 2020) or even by monsoons (Gutierrez and Ponti 2014). Invasive alien insect species may cause much economic damage on agricultural products and cultural heritage, as well as transmit diseases to farm animals and humans (Chapin III et al. 2000, Andersen et al. 2004, Pimentel et al. 2005). As a result of analysing the change in temperature in Korea over the past 106 years (1912-2017), the average temperature in the last 30 years (1988-2017) increased by 1.4°C compared with the early 20th century (1912-1941) (Park and Lee 2019). This shows that we have faced direct climate change in the country, which may accelerate the introduction of invasive alien insect species by directly affecting their development, reproduction and survival. The introduction of invasive alien insect species by monsoons has particularly been affected by climate changes (Gutierrez and Ponti 2014). Invasive alien insect species introduced by monsoons or from other countries have been observed in Korea (Park et al. 2006).

A total of eight invasive alien insect species have been reported in Korea and declared as ecosystem-disturbing species. *Lycormadelicatula* (Hemiptera: Fulgoridae), *Metcalfapruinosa* (Hemiptera: Flatidae) and *Pochaziashantungensis* (Hemiptera: Ricaniidae) are widespread in Korea, where they cause severe ecological damage to crops and trees by piercing-sucking (Han et al. 2008, Park et al. 2009, Kim and Kil 2014, Kim et al. 2015, Choi et al. 2018). *Vespavelutinanigrithorax* (Hymenoptera: Vespidae), which is also widespread in Korea, preys on honeybees, decreasing the earnings of beekeepers and it also harms humans (Choi et al. 2012, Monceau et al. 2013). The ant species *Solenopsisinvicta*, *Anoplolepisgracilipes* and *Linepithemahumile* (all Hymenoptera: Formicidae) are listed amongst the International Union for Conservation of Nature’s (IUCN) 100 most common invasive species (Lowe et al. 2000). These species were probably introduced into Korea via ports and harbours (Jung et al. 2017b, Sung et al. 2018, Lee et al. 2020). When released into natural ecosystems, they are expected to bring severe ecological and economic damage to agriculture, forestry and human health (Pimentel et al. 2005). *Melanoplusdifferentialis* (Differential grasshopper) is a large insect species that competes with any native Korean insect; as it presents diverse feeding habits, it may damage agricultural or forest land (Kang et al. 2020). It is important to continuously monitor the distribution and current status of invasive alien insect species to prevent future problems. Thus, this study can be used as a basis to identify areas that require priority management, based on the nationwide distribution of invasive alien insect species that enter the country through various routes and then settle, thereby, negatively affecting the ecosystem.

## Material and methods

### Study period and target locations

**Figure 1.**
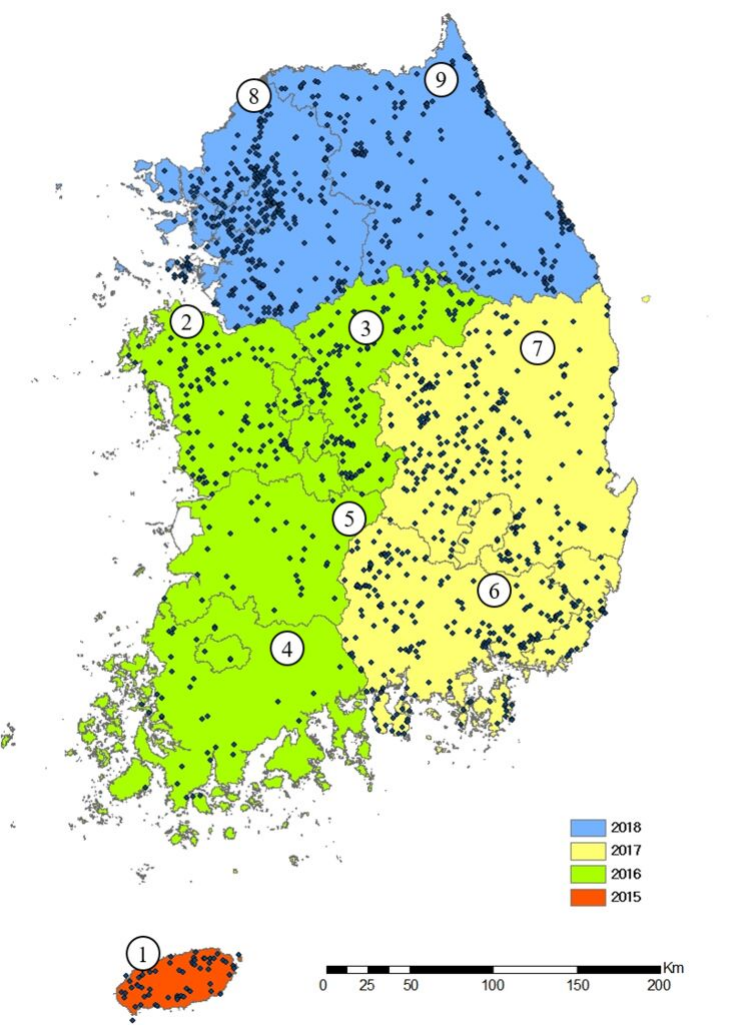
Study sites in Korea. 1. Jeju-do 2. Chungcheongnam-do 3. Chungcheongbuk-do 4. Jeollanam-do 5. Jeollabuk-do 6. Gyeongsangnam-do 7. Gyeongsangbuk-do 8. Gyeonggi-do 9. Gangwon-do

Amongst the invasive alien insect species introduced into Korea, the investigation was conducted from March 2015 to November 2018 focusing on invasive alien insect species found in the natural ecosystem. The survey area was divided into nine regions. The survey was conducted in 67 locations in Jeju Island in 2015, in 337 locations in Chungcheongnam-do, in 311 locations in Chungcheongbuk-do, in 72 locations in Jeollanam-do and in 82 locations in Jeollabuk-do in 2016, in 521 locations in Gyeongsangnam-do and in 560 locations in Gyeongsangbuk-do in 2017 and in 853 locations in Gyeonggi-do and in 446 locations in Gangwon-do in 2018 (Fig. 1). Environmental characteristics of the survey area include grasslands, forests, reservoirs, valleys and marshes, including these environments and surrounding areas such as roadsides, parks, residences and highway rest areas were also investigated (National Institute of Ecology 2015, National Institute of Ecology 2016, National Institute of Ecology 2017, National Institute of Ecology 2018).

### Investigation methods

Presence of insects in each site was investigated in various ways according to the characteristics of the insect classification group. Visual inspection was conducted around the survey area and additional surveys were conducted using tools. An insect net was used for sweeping, brandishing and beating and an insect aspirator was used to collect small insects. In order to collect nocturnal insects, the light traps collection method was used and insects were collected using a method such as malaise traps to catch insects that have a habit of going up when obstacles meet in a similar shape to tents.

### Species richness analysis

We calculated the Dominance Index (DI), Diversity Index (H'), Richness Index (RI) and Evenness Index (EI) for 3,249 locations of the nine regions.

DI (McNaughton 1967) was calculated using the number of locations where the invasive alien insect species were identified. We defined the species with the highest and second highest number of habitats as dominant and subdominant species, respectively, using the following equation:

DI = \begin{varwidth}{50in}
        \begin{equation*}
            (n1+n2)/N
        \end{equation*}
    \end{varwidth}

where n1 is the number of dominant species, n2 is the number of subdominant species and N is the total number of individuals.

H' represents the relationship between species and the number of individuals in a population. It is a measure of species enrichment and uniformity, correlating positively with the species diversity in a population. The following Shannon-Wiener function (H’), developed by Margalef (1958) and then modified by Llyod and Ghelardi (1964), was used (Pielou 1969):

H’ = \begin{varwidth}{50in}
        \begin{equation*}
            -∑(i=1)^s[(ni/n) ln(ni/n)]
        \end{equation*}
    \end{varwidth}

where ni is the number of species and N is the total number of locations.

RI represents the state of a population, based on the number of species and the total number of locations. The higher RI is, the better the environment is in terms of species richness. We calculated RI using Margalef (1958)'s equation:

RI = \begin{varwidth}{50in}
        \begin{equation*}
            (S-1)/(ln (N)) 
        \end{equation*}
    \end{varwidth}

where S is the number of species and N is the total number of locations.

EI represents the species uniformity in a population and is the ratio of the actual index number over the maximum number of the corresponding index. If all the species’ populations are found in the same number of locations, the maximum EI equals one. We calculated EI using the Pielou (1975)'s equation:

EI = \begin{varwidth}{50in}
        \begin{equation*}
            H'/lnS
        \end{equation*}
    \end{varwidth}

where H’ is the DI and S is the total number of species.

### Coordinate analysis

For the 17 species that inhabit the most in Korea, the distribution point is indicated on the map. Using Arc GIS (ver. 10.5), the distribution of 17 species distributed in all nine regions and appear at more than 20 locations is shown on the shapefile map of the Korean Peninsula.

## Results

### Species composition

Over the four years, we identified 63 species in 43 families and nine orders of invasive alien insect species inhabiting the 3,249 locations in Korea. Hemiptera exhibited the highest species diversity, with 20 species in 1,972 locations, followed by 12 species of Coleoptera in 676 locations, 12 species of Lepidoptera in 268 locations, five species of Diptera in 86 locations, five species of Blattodae in 97 locations and four species of Hymenoptera in 133 locations (Table 1).

### Composition of regional taxa

In the Jeju-do region, we found seven species in six families and four orders of invasive alien insect species. Seven species were found in 67 locations. In the Chungcheongnam-do region, we identified 20 species in 14 families and six orders in 337 locations. In the Chungcheongbuk-do area, we identified 10 species in eight families and five orders in 311 locations. In the Jeollanam-do region, we identified 15 species in 13 families and six orders in 72 locations. In the Jeollabuk-do region, we identified 16 species in 11 families and six orders in 82 locations. In the Gyeongsangnam-do area, we identified 27 species in 20 families and six orders in 521 locations. In the Gyeongsangbuk-do region, we identified 28 species in 21 families and six orders in 560 locations. In the Gyeonggi-do area, we identified 45 species in 33 families and eight orders in 853 locations. In the Gangwon-do region, we identified 28 species in 19 families and seven orders in 446 locations (Table 2).

### Species richness analysis

As a result of Species richness analysis, the DI, H’, EI and RI showed different high indices depending on the region. The Jeju-do area showed the highest DI (0.8). As for the H', the Gyeongsnagnam-do region showed the highest value (2.8). As for the EI, Jeollanam-do, Chungcheongbuk-do and Gyeongsangnam-do showed values higher than (0.9). Finally, regarding RI, Gyeonggi-do showed the highest value (6.5) (Table 3).

### Distribution of the invasive alien insect species in Korea

Amongs the 63 invasive alien insects found in Korea, 17 invasive alien insects found in more than 20 locations were indicated on the map. *Corythuchamarmorata* appeared in the highest number of locations (508 locations, Fig. 2a), followed by *Lycormadelicatula* (447 locations, Fig. 2b). *Ophraellacommuna* (360 locations, Fig. 2c), *Metcalfapruinosa* (356 locations, Fig. 2d), *Pochaziashantungensis* (311 locations, Fig. 2e), *Corythuchaciliate* (215 locations, Fig. 2f), *Vespavelutinanigrithorax* (117 locations, Fig. 3a), *Euremamandarina* (117 locations, Fig. 3b) *Lissorhoptrusoryzophilus* (96 locations, Fig. 3c), *Hyperapostica* (90 locations, Fig. 3d), *Hyphantriacunea* (81 locations, Fig. 3e), *Ceutorhynchusobstrictus* (78 locations, Fig. 3f), *Leptoglossusoccidentalis* (74 locations, Fig. 4a), *Hermetiaillucens* (71 locations, Fig. 4b), *Blattellagermanica* (52 locations, Fig. 4c), *Hyperapunctate* (43 locations, Fig. 4d) and *Reticulitermessperatus* (35 locations, Fig. 4e) (Figs 2, 3, 4).

17 invasive alien insect species shown on the map was classified as follows: six species of Hemiptera, five species of Coleoptera, two species of Lepidoptera, two species of Blattodea, one species of Diptera and one species of Hymenoptera.

### Types of habitat

The habitats of the invasive alien insect species in Korea were diverse, including roadsides, farmlands, parks, forests and residential areas. Amongst these habitats, the species were most frequently found on roadsides (31.3%), followed by farmlands (18.3%), parks (17.5%), forests (16.6%), residential areas (8.0%), orchards (1.7%), watersides (6.3%) and others (0.4%) (Fig. 5a).

In the forests, the habitat of invasive alien insect species was identified in the order of mixed forest (43.3%), forest roads (35.4%), mountainous districts (21.1%) and farm (0.3%). The waterside includes riversides (51.1%), wetlands (35.6%), reservoirs (11.3%) and valleys (2.1%) and the most invasive alien insect species were found on the riverside. Additionally, there are other areas (45.0%), beaches (25.0%), rest areas (20.0%) and camping sites (10.0%) (Fig. 5b).

## Conclusion

From 2015 and 2018, we identified 63 species in 43 families and nine orders of invasive alien insect species in the Korean ecosystems. We analysed the distribution and diversity of the invasive alien insect species from nine regions and compared the DI, H’, RI and EI of the species in each region.

Park (2010) reported 102 species of invasive alien insect species in Korea between 1990 and 2012, whereas Hong et al. (2012) reported 171 species between 1996 and 2014. The number of species herein obtained differs from those reported in the two studies mentioned above, possibly because they included pests for biological control, research or pollination (which are usually excluded from facilities, horticulture and prohibited items), whereas the present study focused on the invasive species in natural Korean ecosystems.

Depending on the local temperature and geographical impact, invasive alien insect species found between regions may differ. Due to these effects, it seems that the species richness analysis results were different for each region. It seems that the regions with the highest DI, H', RI and EI values appeared differently due to the influence of urbanisation and geographical isolation. Marques et al. (2020) reported that urbanisation promotes the adaptation of alien species to the new habitats. In the case of Korea, urbanisation has progressed rapidly due to the spread of population to Gyeonggi-do and the development of urban areas due to the problem of population growth and traffic congestion in Seoul (Kim et al. 2021). This is supported by our study, as the Gyeonggi-do region (Table 2), which includes the cities Seoul and Incheon and it is the most urbanised of our study sites, had the highest number of invasive alien insects (45 species). Conversely, we identified only seven invasive alien insect species in the Jeju-do region. Its geographical isolation may have resulted in the introduction of less invasive insect species in the Jeju-do region than in the other areas. However, due to climate changes, an increase in flying pests and a revival in tourism may increase the number of invasive alien insect species in the Jeju-do region (Jung et al. 2017a).

The habitats of the invasive alien insect species introduced via various routes can be ranked in descending order as follows: roadsides, farmlands and parks. More than 83% of the invasive alien insect species were found on the roadsides, farmlands and parks. Amongst them, more than 31% of the invasive alien insect species were found on the roadside. This could be explained by the fact that invasive alien insect species are often introduced via transportation of freight and shipping containers (Thomas et al. 2017), increasing the probability of invasive alien insects spreading to roadside areas. Furthermore, the vegetation planted on the roadside feed insect species, promotes migration, serves as shelter, maintains ecological diversity and provides a suitable environment for insect species, which can be explained by the high habitat of invasive alien insects on the roadside (Muñoz et al. 2015, Marques et al. 2020).

Climate changes are increasing the overall temperature during summer and the lowest temperature during winter in Korea. As temperature directly affects the development, reproduction and survival of insect species (Bale et al. 2002), the higher temperatures in Korea are likely to increase the insect species’ probability of surviving winter, as well as their reproductive capacity and accelerate their development, leading to their explosive spread in Korea (Musolin 2007, Hong et al. 2019). In particular, the invasion and adaptation of invasive alien insect species are easier in port regions than in other locations (Gutierrez and Ponti 2014). As temperatures in Korea increase, invasive alien insect species, such as *Solenopsisinvicta*, *Anoplolepisgracilipes*, *Pochaziashantungensis* and *Vespavelutinanigrithorax* are likely to spread across the country (Kim et al. 2015, Jung et al. 2017b, Byeon et al. 2020). Therefore, it is necessary to strengthen port quarantine procedures and to promptly respond to the introduction of invasive alien insect species in Korea.

Therefore, the unintentional introduction of invasive alien insect species in Korea must be carefully monitored. Additional investigations are needed to establish standard procedures and to prevent a further spread of the invasive alien insect species in the country. Each region of Korea should also constantly remove and manage the invasive alien insect species that may disturb the ecological equilibrium.

## Appendix

List of invasive alien insect species found in the natural ecosystem (Table 4).

## Figures and Tables

**Figure 2a.**
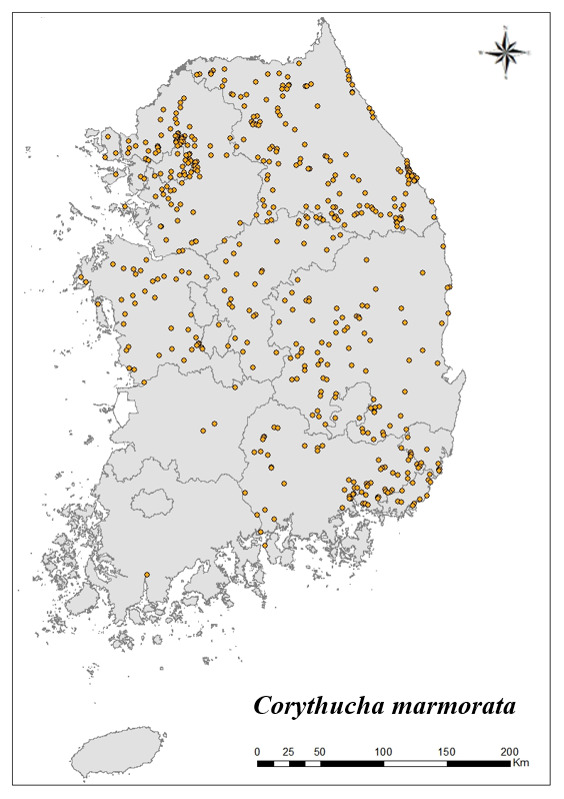
1. *Corythuchamarmorata*

**Figure 2b.**
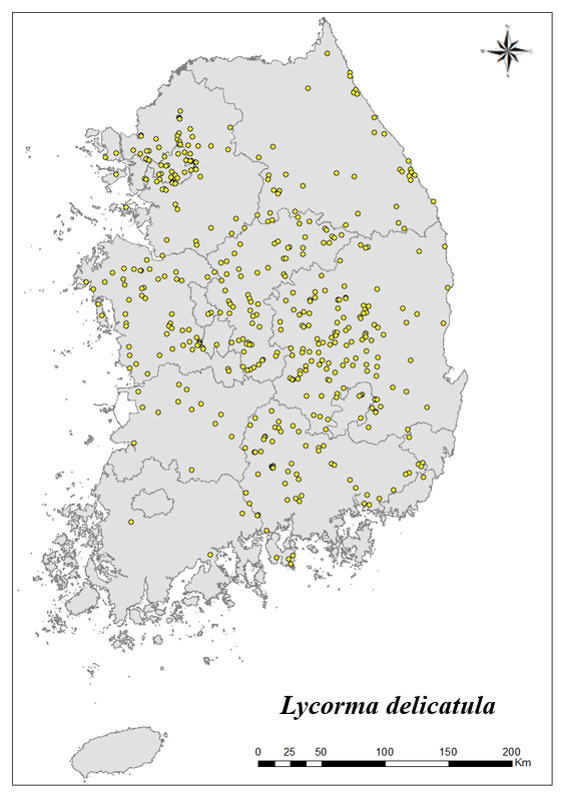
2. *Lycormadelicatula*

**Figure 2c.**
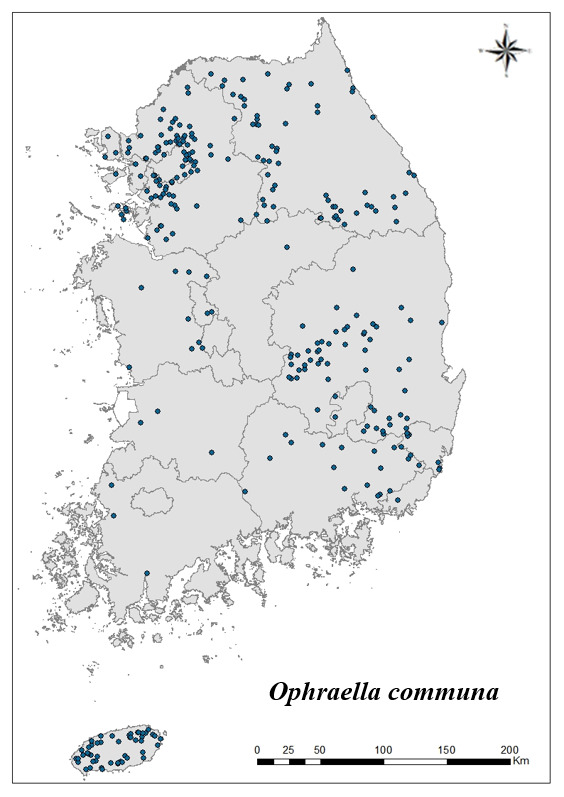
3. *Ophraellacommuna*

**Figure 2d.**
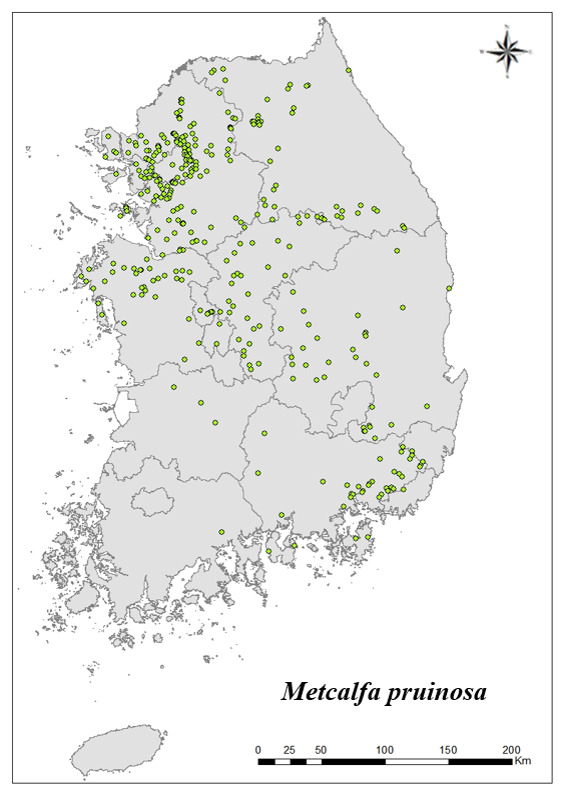
4. *Metcalfapruinosa*

**Figure 2e.**
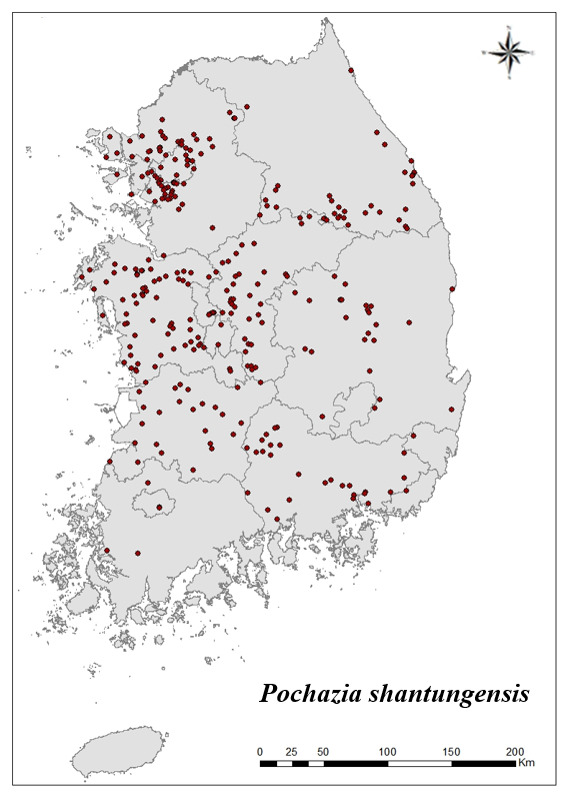
5. *Pochaziashantungensis*

**Figure 2f.**
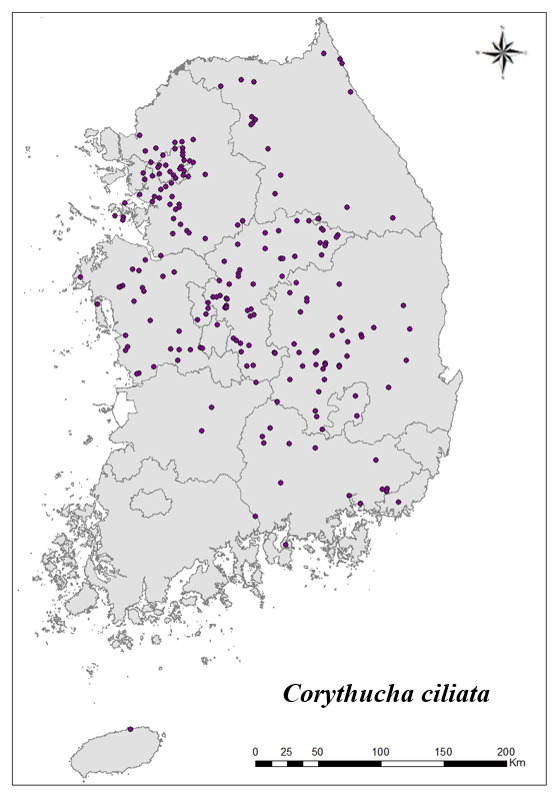
6. *Corythuchaciliata*

**Figure 3a.**
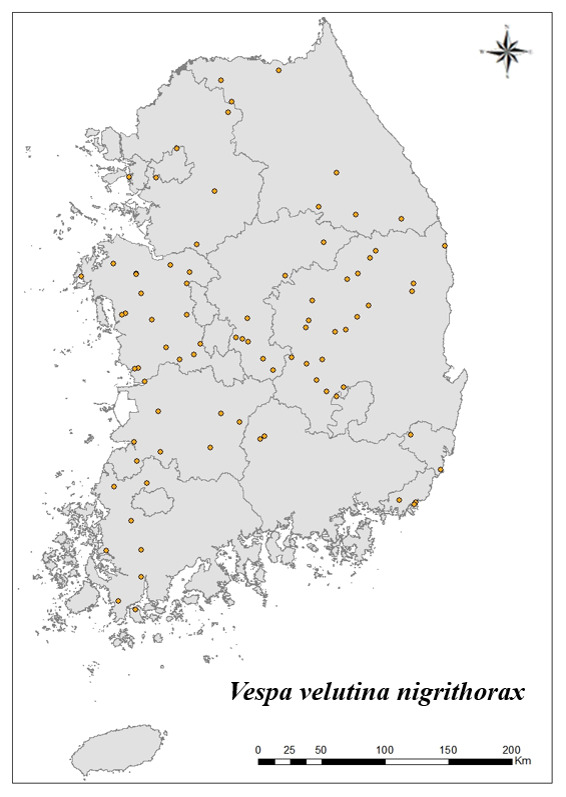
7. *Vespavelutinanigrithorax*

**Figure 3b.**
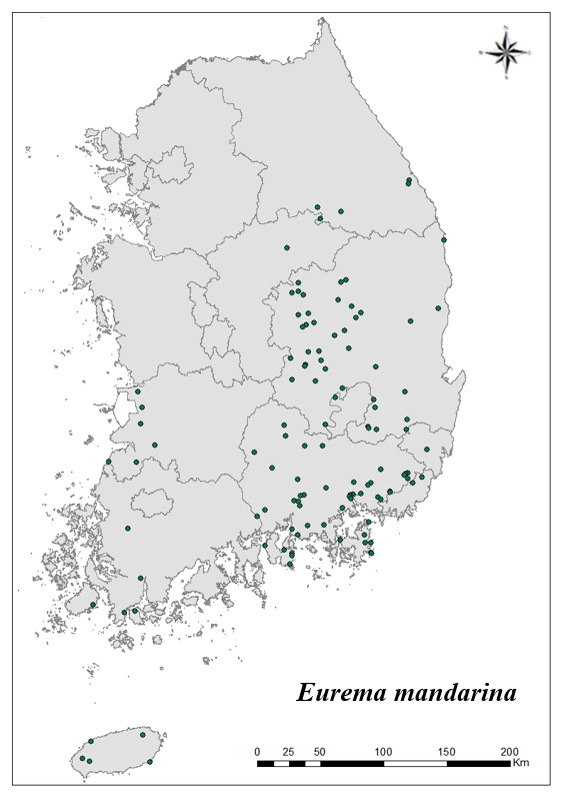
8. *Euremamandarina*

**Figure 3c.**
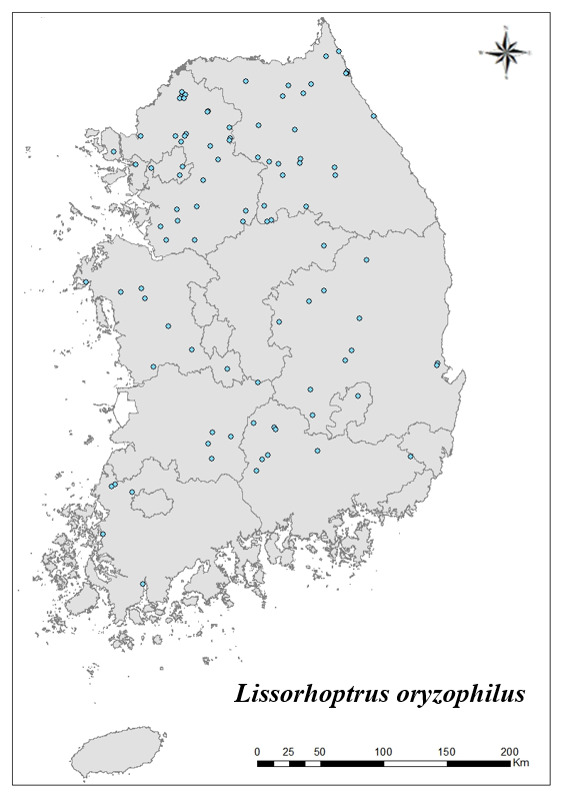
9. *Lissorhoptrusoryzophilus*

**Figure 3d.**
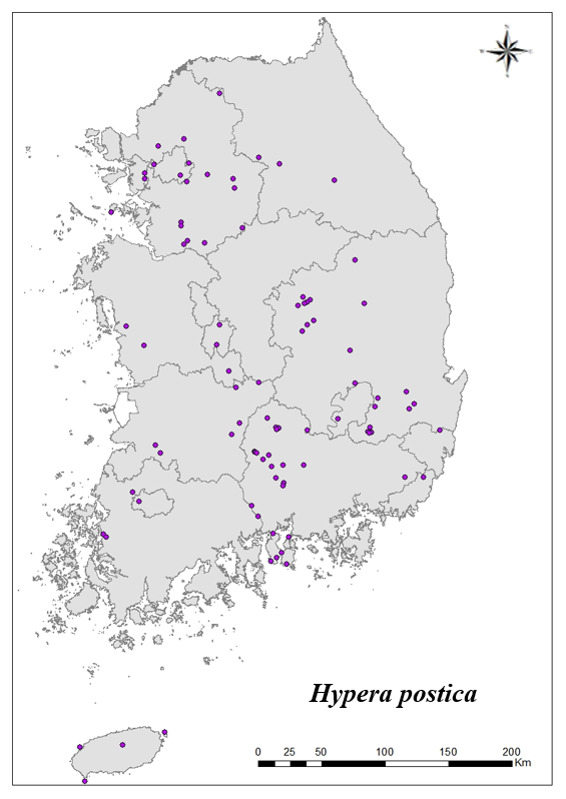
10. *Hyperapostica*

**Figure 3e.**
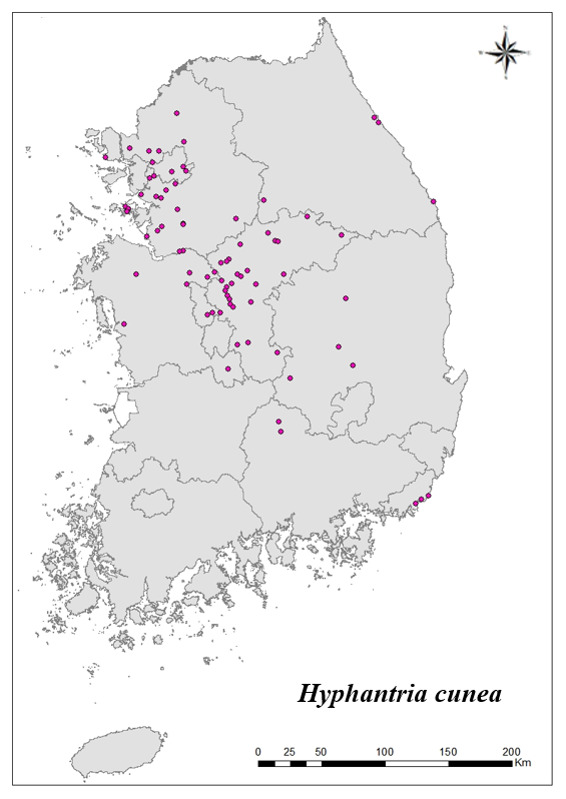
11. *Hyphantriacunea*

**Figure 3f.**
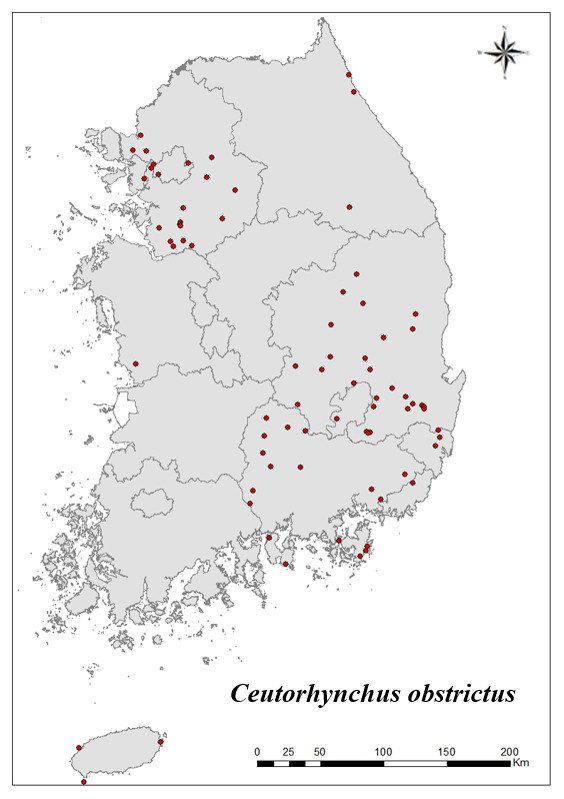
12. *Ceutorhynchusobstrictus*

**Figure 4a.**
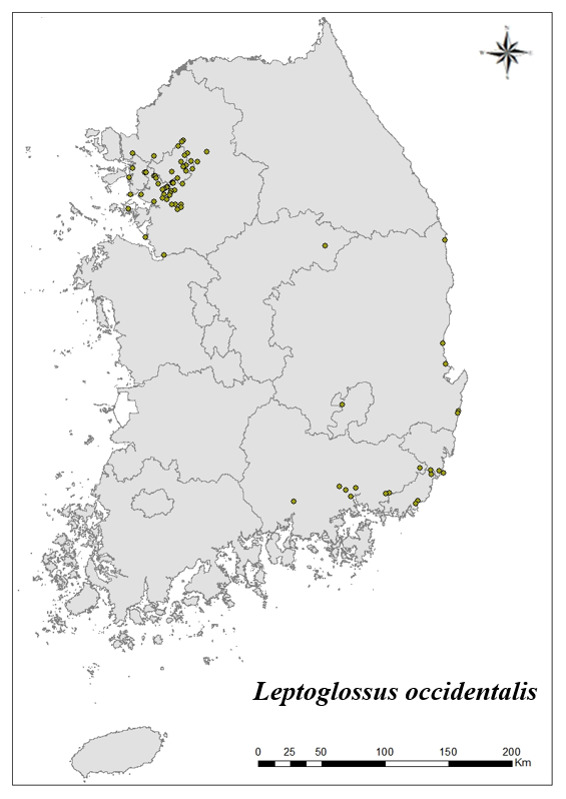
13. *Leptoglossusoccidentalis*

**Figure 4b.**
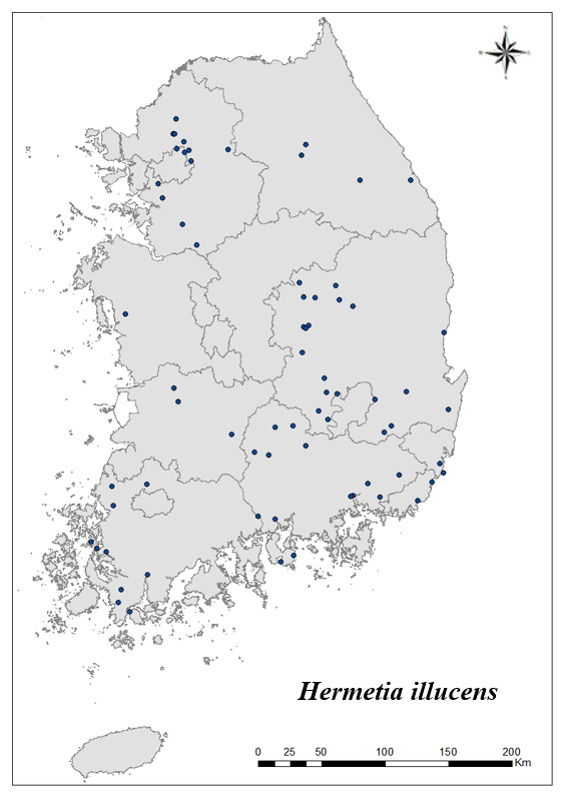
14. *Hermetiaillucens*

**Figure 4c.**
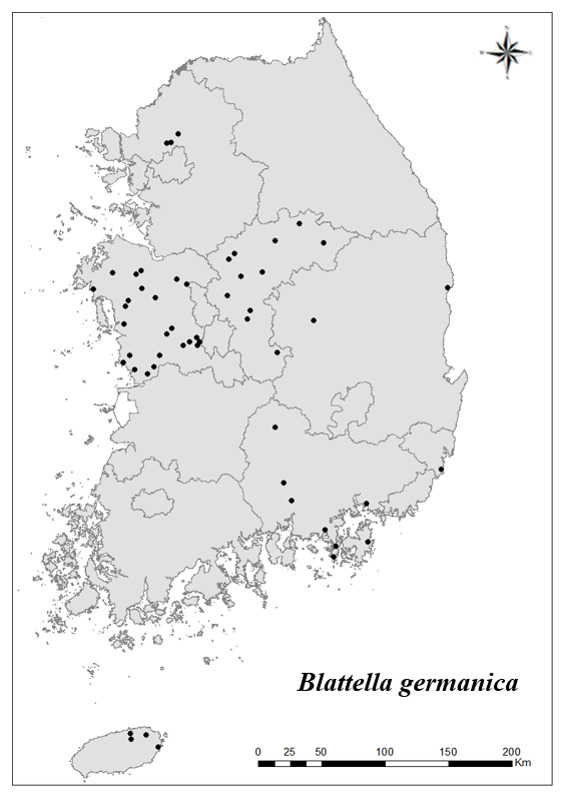
15. *Blattellagermanica*

**Figure 4d.**
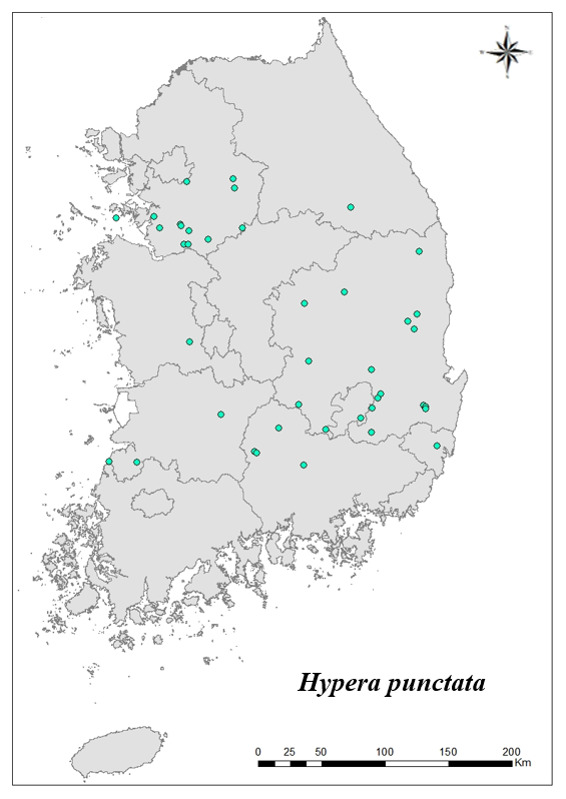
16. *Hyperapunctata*

**Figure 4e.**
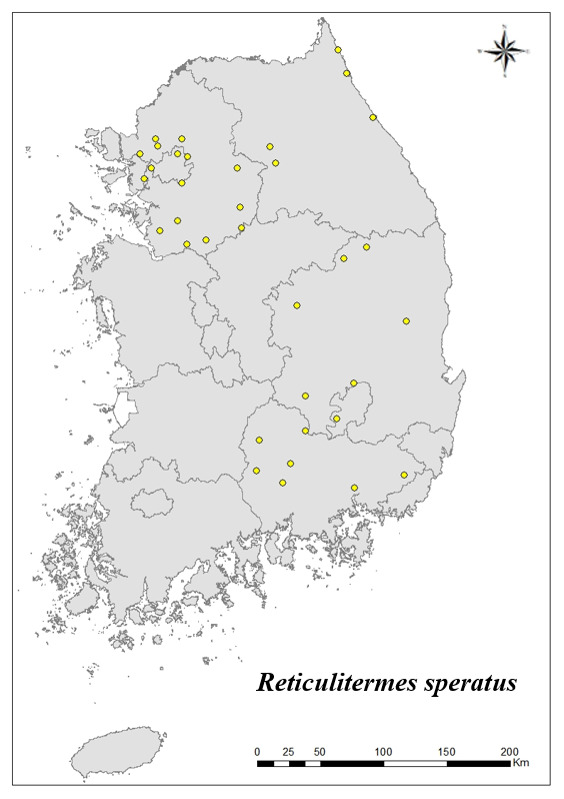
17. *Reticulitermessperatus*

**Figure 5a.**
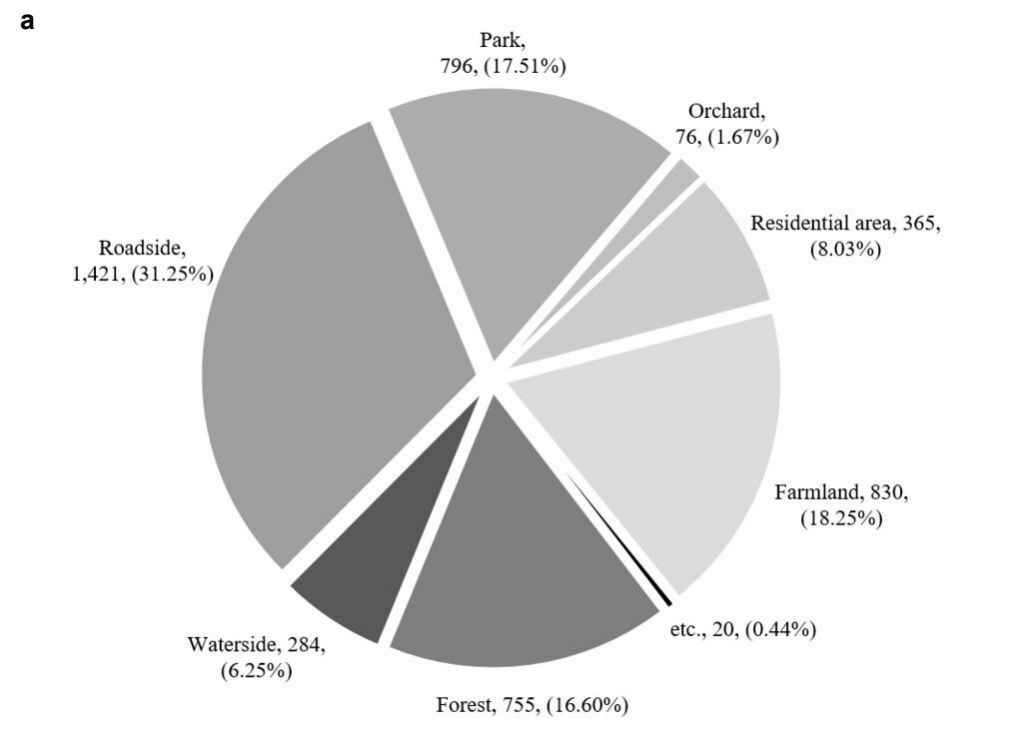
Proportion of each habitat

**Figure 5b.**
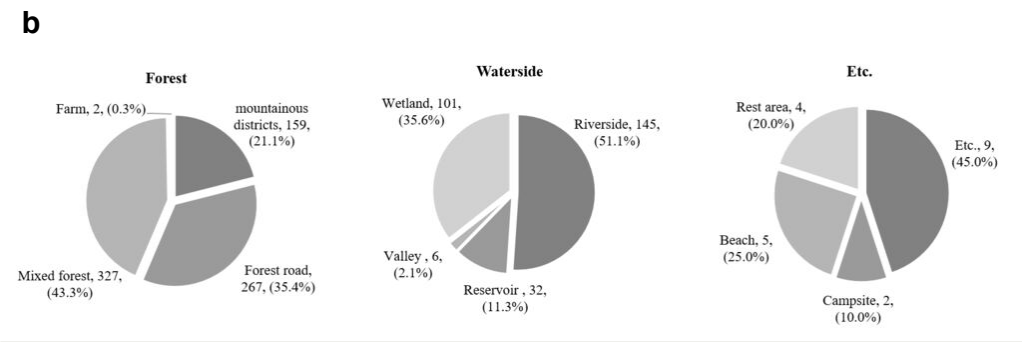
Proportion of forests, watersides and other areas

**Table 1.** Distribution of the invasive alien insect species found in Korea.

**Order**	**No. families**	**No. species**	% **of species**	No. of research Site*	% of research Site*
Blattodea	3	5	7.9	97	3.0
Coleoptera	6	12	19.1	676	20.8
Diptera	5	5	7.9	86	2.7
Hemiptera	14	20	31.8	1,972	60.7
Hymenoptera	3	4	6.4	133	4.1
Lepidoptera	9	12	19.1	268	8.3
Odonata	1	1	1.6	1	0.0
Psocodea	1	1	1.6	2	0.1
Thysanoptera	1	3	4.8	14	0.4
9	43	63	100.0	3,249	100.0

*: Number of locations where the invasive alien insect species were observed.

**Table 2.** Regional distribution of the invasive alien insect species in Korea.

**Survey area**	**Order**	**No. of families**	**No. of species**	% **of species**	**No. of research sites**	% **of research sites**	**No. of cities and counties**
Jeju-do (2015)	Blattodea	1	1	14.3	4	6.0	2
	Coleoptera	2	3	42.9	55	82.1	2
	Hemiptera	1	1	14.3	1	1.5	1
	Lepidoptera	2	2	28.6	7	10.5	2
Subtotal no.	4	6	7	100.0	67	100.0	
Chungcheongnam-do (2016)	Blattodea	2	2	10.0	25	7.4	14
	Coleoptera	2	6	30.0	27	8.0	16
	Diptera	2	2	10.0	2	0.6	2
	Hemiptera	5	6	30.0	249	73.9	17
	Hymenoptera	2	3	15.0	25	7.4	14
	Lepidoptera	1	1	5.0	9	2.7	4
Subtotal no.	6	14	20	100.0	337	100.0	
Chungcheongbuk-do (2016)	Blattodea	2	2	20.0	11	3.5	11
	Coleoptera	1	1	10.0	52	16.7	10
	Hemiptera	4	5	50.0	212	68.2	11
	Hymenoptera	1	1	10.0	8	2.6	4
	Lepidoptera	1	1	10.0	28	9.0	11
Subtotal no.	5	8	10	100.0	311	100.0	
Jeollanam-do (2016)	Blattodea	1	1	6.3	2	2.8	2
	Coleoptera	2	4	25.0	18	25.0	11
	Diptera	1	1	6.3	10	13.9	8
	Hemiptera	6	6	37.3	25	34.7	15
	Hymenoptera	3	3	18.8	12	16.7	11
	Lepidoptera	1	1	6.3	5	6.9	5
Subtotal no.	6	14	16	100.0	72	100.0	
Jeollabuk-do (2016)	Coleoptera	2	4	25.0	16	19.5	9
	Diptera	1	1	6.3	3	3.7	3
	Hemiptera	5	8	50.0	48	58.5	14
	Hymenoptera	1	1	6.3	8	9.8	8
	Lepidoptera	1	1	6.3	6	7.3	5
	Odonata	1	1	6.3	1	1.2	1
Subtotal no.	6	11	16	100.0	82	100.0	
Gyeongsangnam-do (2017)	Blattodea	2	2	7.4	14	2.7	11
	Coleoptera	2	5	18.5	91	17.5	17
	Diptera	1	1	3.7	19	3.7	12
	Hemiptera	8	11	40.7	272	52.2	19
	Hymenoptera	2	2	7.4	42	8.1	15
	Lepidoptera	5	6	22.2	83	15.9	17
Subtotal no.	6	20	27	100.0	521	100.0	
Gyeongsangbuk-do (2017)	Blattodea	2	2	7.1	9	1.6	8
	Coleoptera	2	5	17.9	137	24.5	20
	Diptera	1	1	3.6	21	3.8	13
	Hemiptera	7	9	32.1	311	55.5	21
	Hymenoptera	3	3	10.7	24	4.3	15
	Lepidoptera	6	8	28.6	58	10.4	16
Subtotal no.	6	21	28	100.0	560	100.0	
Gyeonggi-do (2018)	Blattodea	3	4	8.9	27	3.2	17
	Coleoptera	6	11	24.4	186	22.0	31
	Diptera	4	4	8.9	21	2.5	15
	Hemiptera	10	13	28.9	552	65.3	32
	Hymenoptera	1	1	2.2	6	0.7	6
	Lepidoptera	7	8	17.8	46	5.4	21
	Psocodea	1	1	2.2	2	0.2	1
	Thysanoptera	1	3	6.7	6	0.7	3
Subtotal no.	8	33	45	100.0	853	100.0	
Gangwon-do (2018)	Coleoptera	2	5	17.9	94	21.0	18
	Diptera	2	2	7.1	10	2.2	8
	Hemiptera	6	9	32.1	296	66.2	18
	Hymenoptera	2	2	7.1	8	1.8	7
	Lepidoptera	5	6	21.4	26	5.8	9
	Thysanoptera	1	3	10.7	8	1.8	6
Subtotal no.	7	19	28	100.0	446	100.0	

**Table 3.** Results of the species richness analysis of the invasive alien insect species in each region.

**Survey area**	**DI**	**H**’	**EI**	**RI**
Jeju-do	0.8	1.1	0.6	1.4
Chungcheongnam-do	0.4	2.3	0.8	3.3
Chungcheongbuk-do	0.4	2.1	0.9	1.6
Jeollanam-do	0.3	2.5	0.9	3.3
Jeollabuk-do	0.4	2.3	0.8	3.4
Gyeongsangnam-do	0.3	2.8	0.9	4.2
Gyeongsangbuk-do	0.4	2.6	0.8	4.3
Gyeonggi-do	0.3	2.7	0.7	6.5
Gangwon-do	0.5	2.3	0.7	4.4

**Table 4.** List of invasive alien insect species in Korea.

**Scientific name**	**JJ**	**CN**	**CB**	**JN**	**JB**	**GN**	**GB**	**GG**	**GW**	**Total**
**Order Blattodea**										
**Family Blattidae**										
*Periplanetajaponica* Karny, 1908		1						2		3
*Periplanetafuliginosa* (Serville, 1839)								6		6
*Periplanetaamericana* (Linnaeus, 1758)			1							1
**Family Ectobiidae**										
*Blattellagermanica* (Linnaeus, 1767)	4	24	11			9	2	3		52
**Famiy Rhinotermitidae**										
*Reticulitermessperatus* Morimoto,1968				2		5	7	16	5	35
**Order Coleoptera**										
**Family Anthribidae**										
*Araecerusfasciculatus* (DeGeer, 1775)								1		1
**Family Chrysomelidae**										
*Callosobruchuschinensis* (Linnaeus, 1758)								1		1
*Ophraellacommuna* LeSage, 1986	48	12	52	6	2	26	57	94	63	360
**Family Curculionidae**										
*Ceutorhynchusobstrictus* (Marsham, 1802)	3	1				24	27	21	2	78
*Hyperapostica* (Gyllenhal, 1813)	4	4		5	6	27	22	19	3	90
*Hyperapunctata* Fabricius, 1775		1			3	7	19	13		43
*Hyperarumicis* (Linnaeus, 1758)		1						1		2
*Lissorhoptrusoryzophilus* Kuschel, 1952		8		7	5	7	12	32	25	96
*Listroderescostirostris* Schoenherr, 1826									1	1
**Family Dermestidae**										
*Attagenusunicolorjaponicus* Reitter, 1877								2		2
**Family Dryophthoridae**										
*Sitophilusoryzae* (Linnaeus, 1763)								1		1
**Family Tenebrionidae**										
*Triboliumcastaneum* (Herbst, 1797)								1		1
**Order Diptera**										
**Family Cecidomyiidae**										
*Obolodiplosisrobiniae* (Haldeman, 1847)								4	6	10
**Family Drosophilidae**										
*Drosophilamelanogaster* Meigen, 1830								1		1
**Family Psychodidae**										
*Psychodaalternata* Say, 1824								3		3
**Family Agromyzidae**										
*Liriomyzatrifolii* (Burgess, 1880)		1								1
**Family Stratiomyidae**										
*Hermetiaillucens* (Linnaeus, 1758)		1		10	3	19	21	13	4	71
**Order Hemiptera**										
**Family Aleyrodidae**										
*Bemisiatabaci* (Gennadius, 1889)								4	3	7
*Trialeurodesvaporariorum* (Westwood, 1856)							1	1	1	3
**Family Anthocoridae**										
*Orius* (Heterorius) *strigicollis* (Poppius, 1915)								2		2
**Family Aphididae**										
*Macrosiphumeuphorbiae* Thomas, 1878								1		1
**Family Coreidae**										
*Leptoglossusoccidentalis* Heidemann, 1910						15	6	53		74
**Family Delphacidae**										
*Laodelphaxstriatellus* (Fallén, 1826)						10	2	3	1	16
*Nilaparvatalugens* (Stål, 1854)		1				3				4
*Sogatellafurcifera* (Horváth, 1899)						8	3	7	2	20
**Family Flatidae**										
*Metcalfapruinosa* (Say, 1830)		42	29	2	2	38	35	164	44	356
*Salurnismarginella* (Guérin-Méneville, 1829)					2					2
**Family Fulgoridae**										
*Lycormadelicatula* (White, 1845)		60	67	7	11	63	121	78	40	447
**Family Phylloxeridae**										
*Aphanostigmaiaksuiense* (Kishida, 1924)								1		1
**Family Issidae**										
*Dentatissusdamnosus* (Chou & Lu, 1985)						1				1
**Family Monophlebidae**										
*Iceryapurchasi* Maskell, 1878						1				1
**Family Machaerotidae**										
*Hindoloidesbipunctatus* (Haupt, 1924)					1					1
**Family Ricaniidae**										
*Pochaziashantungensis* Chou & Lu, 1977		73	37	10	25	29	26	76	35	311
*Ricaniaspeculum* (Walker, 1851)					1					1
**Family Scutelleridae**										
*Cantaoocellatus* (Thunberg, 1784)				1						1
**Family Tingidae**										
*Corythuchamarmorata* (Uhler, 1878)		42	33	2	3	88	80	109	151	508
*Corythuchaciliata* (Say, 1832)	1	31	47	2	3	16	37	60	18	215
**Order Hymenoptera**										
**Family Apidae**										
*Bombusterrestris* (Linnaeus, 1758)				2		2	2		1	7
**Family Formicidae**										
*Formicayessensis* Wheeler, 1913		5		1			1			7
*Monomoriumpharaonis* (Linnaeus, 1758)		2								2
**Family Vespidae**										
*Vespavelutinanigrithorax* du Buysson, 1905		18	8	9	8	40	21	6	7	117
**Order Lepidoptera**										
**Family Crambidae**										
*Cnaphalocrocismedinalis* Guenée, 1854						8	2	1		11
**Family Erebidae**										
*Hyphantriacunea* (Drury, 1773)		9	28			5	4	31	4	81
**Family Lycaenidae**										
*Curetisacuta* Moore, 1877						12	1			13
*Lampidesboeticus* (Linnaeus, 1767)	2					3	1	1		7
**Family Noctuidae**										
*Mythimnaseparata* (Walker, 1865)							1		1	2
*Peridromasaucia* (Hübner, 1808)								1		1
*Spodopteraexigua* (Hübner, 1808)							5	4	7	16
**Family Papilionidae**										
*Papiliohelenus* Linnaeus, 1758						2				2
**Family Pieridae**										
*Euremamandarina* (de l’Orza, 1869)	5			5	6	53	43		5	117
**Family Psychidae**										
*Eumetavariegata* (Snellen, 1879)								1	3	4
**Family Pyralidae**										
*Plodiainterpunctella* Hübner, 1810								2		2
**Family Tortricidae**										
*Grapholitamolesta* (Busk, 1916)							1	5	6	12
**Order Odonata**										
**Family Libellulidae**										
*Brachydiplaxchalybeaflavovittata* Ris, 1911					1					1
**Order Psocodea**										
**Family Liposcelididae**										
*Liposcelisentomophila* (Enderlein, 1907)								2		2
**Order Thysanoptera**										
**Family Thripidae**										
*Frankliniellaoccidentalis* (Pergande, 1895)								3	5	8
*Thripspalmi* Karny, 1925								2	1	3
*Thripstabaci* Lindeman, 1889								1	2	3
**Total**	67	337	313	71	82	521	560	853	446	3,249

- JJ: Jeju-do; CN: Chungcheongnam-do; CB: Chungcheongbuk-do; JN: Jeollanam-do; JB: Jeollabuk-do; GN: Gyeongsangnam-do; GB: Gyeongsangbuk-do; GG: Gyeonggi-do; GW: Gangwon-do
